# Deep learning and artificial intelligence in radiology: Current applications and future directions

**DOI:** 10.1371/journal.pmed.1002707

**Published:** 2018-11-30

**Authors:** Koichiro Yasaka, Osamu Abe

**Affiliations:** 1 Department of Radiology, The Institute of Medical Science, The University of Tokyo, Tokyo, Japan; 2 Department of Radiology, Graduate School of Medicine, The University of Tokyo, Tokyo, Japan

Radiological imaging diagnosis plays important roles in clinical patient management. Deep learning with convolutional neural networks (CNNs) is recently gaining wide attention for its high performance in recognizing images. If CNNs realize their promise in the context of radiology, they are anticipated to help radiologists achieve diagnostic excellence and to enhance patient healthcare. Here, we discuss very recent developments in the field, including studies published in the current *PLOS Medicine* Special Issue on Machine Learning in Health and Biomedicine, with comment on expectations and planning for artificial intelligence (AI) in the radiology clinic.

Chest radiographs are one of the most utilized radiological modalities in the world and have been collected into a number of large datasets currently available to machine learning researchers. In this Special Issue, three groups of researchers applied deep learning to radiological imaging diagnosis using this modality. In the first, Pranav Rajpurkar and colleagues found that deep learning models detected clinically important abnormalities (e.g., edema, fibrosis, mass, pneumonia, and pneumothorax) on chest radiography, at a performance level comparable to practicing radiologists [1]. In a similar study, Andrew Taylor and colleagues developed deep learning models that detected clinically significant pneumothoraces on chest radiography with excellent performance on data from the same site—with areas under the receiver operating characteristic curve (AUC) of 0.94–0.96 [2]. Meanwhile, Eric Oermann and colleagues investigated how well deep learning models that detected pneumonia on chest radiography generalized across different hospitals. They found that models trained on pooled data from sites with different pneumonia prevalence performed well on new pooled data from these same sites (AUC of 0.93–0.94) but significantly less well on external data (AUC 0.75–0.89); additional analyses supported the interpretation that deep learning models diagnosing pneumonia on chest radiography are able to exploit confounding information that is associated with pneumonia prevalence [3]. Also in this Special Issue, Nicholas Bien and colleagues applied deep learning techniques to detect knee abnormalities on magnetic resonance (MR) imaging and found that the trained model showed near-human-level performance [4]. Taking these four studies together, we can interpret that deep learning is currently able to diagnose a number of conditions using radiological data, but such diagnostic models may not be robust to a change in location.

These Special Issue studies join a growing number of applications of deep learning to radiological images from various modalities that can aid with detection, diagnosis, staging, and subclassification of conditions. Cerebral aneurysms can be detected on MR angiography with sensitivity/false-positive findings of 0.70/0.26 (low false positive model) or 0.94/2.90 (high sensitivity model) [5]. Liver masses can be classified into five categories (from classical hepatocellular carcinoma as category A to liver cyst as category E) using a combination of dynamic contrast enhanced-computed tomography (CT) images [6]. The staging of liver fibrosis on gadoxetic acid–enhanced MR images is also possible. For this application, the deep learning model was trained using histopathologically evaluated liver fibrosis stages as reference data. The model was able to stage liver fibrosis, with an AUC of approximately 0.85 [7]. Other developments within oncology are appearing in the literature. The genomic status of gliomas can be estimated by deep learning models trained on MR images that can predict isocitrate dehydrogenase 1 mutation status and O6-methylguanine-DNA methyltransferase promotor methylation status with an accuracy of 0.94 and 0.83, respectively [8]. And according to a final study from this Special Issue, a cancer patient’s prognosis may also be estimated with deep learning. Hugo Aerts and colleagues report that their model was able to stratify patients with non–small cell lung cancer into low- and high-mortality risk groups using standard-of-care CT images [9].

Other deep learning applications within radiology can assist with image processing at earlier stages. Segmentation of organs or tissues within images is possible with deep learning, as in a recent *PLOS ONE* research article in which Andrew Grainger and colleagues report the development of a model that quantifies visceral and subcutaneous fat from MR images of the mouse abdomen [10]. In another clever application, Fang Liu and colleagues developed a deep learning model to generate CT images from MR images. They used these images for attenuation correction in reconstructing positron emission tomography (PET) images in PET-MR examinations in which bone information is difficult to obtain. Using the generated pseudo-CT images, less PET reconstruction error was achieved compared with conventional MR imaging–based attenuation correction approaches in brain PET-MR examinations [11].

Deep learning models, if validated for performance, offer several potential benefits to clinicians and patients, starting as early as the education of radiologists. Models trained using images labeled by experienced radiologists, specialty radiologists, and/or histopathological reports may in the future provide a training tool to help trainees or general radiologists to gain competence and confidence in difficult diagnoses. Deep learning models may also help trained radiologists achieve higher interrater reliability throughout their years in clinical practice. In this Special Issue, Bien and colleagues demonstrated that the Fleiss’ kappa measure of interrater reliability for detecting anterior cruciate ligament tear, meniscal tear, and abnormality were higher with model assistance than without [4].

Second, deep learning models may help shoulder the increasing workload in radiology. Newer imaging modalities such as CT and MR can provide more detailed information with thinner images and/or multiple series of images, and the time required to collect these images is shorter than before. Therefore, the number of images collected in each examination is increasing, whereas the number of radiologists who interpret these images is not. Radiologist fatigue can be alleviated if deep learning models can undertake supportive tasks 24 hours a day. Third, deep learning models can also be used to alert radiologists and physicians to patients who require urgent treatment, as in the application described by Taylor and colleagues in the detection of pneumothorax [2]. In a conceptually related application, Luciano Prevedello and colleagues developed a model that detects critical findings (hemorrhage, mass effect, and hydrocephalus) with an AUC of 0.91 on unenhanced head CT [12]. In more granular applications, models that can sort imaging findings according to urgency may optimize radiology workflow. Finally, deep learning models trained to predict histopathological findings based on noninvasive images, such as the models described above that use MR to stage liver fibrosis [7], may help in reducing the risk of complications from invasive biopsy.

We should also acknowledge that deep learning has certain limitations. First, the features and calculations that deep learning models use to make a classification are challenging to interpret. Therefore, when the judgment of physicians or radiologists differ from that of trained models, the discrepancy cannot be resolved by discussion. A potential compromise exists in certain other AI strategies, such as decision trees, that are fully interpretable—however, at this time, a trade-off relationship between interpretability and performance exists. Some technical investigators are working to develop “explainable AI,” with the high performance of deep learning in interpretable models (https://www.darpa.mil/program/explainable-artificial-intelligence), but this has not been fully achieved at the present time. Gradient-weighted Class Activation Mapping is a currently available technique used to visualize the regions of images that were of key importance to deep learning models’ prediction [13]. In this Special Issue, Aerts and colleagues, who developed the network for mortality risk stratification from standard-of-care CT images of non–small cell lung cancer patients, used this technique. The trained network was found to fixate on the interface between the tumor and stroma (lung parenchyma or pleura) [9]. Though this technique allows us to know where the important features exist, there remains a problem; the method does not explicitly show what the important features are. However, with further advancement of these techniques, it may become possible to interpret how AI reaches a decision and even derive new pathophysiologic knowledge from trained AI models.

In addition to limited interpretability, deep learning models—like machine learning models generally—are prone to overfitting and do not necessarily show consistent performance when analyzing data not used during training. To overcome the overfitting problem, a large amount of image data accompanied with valid reference labels (i.e., clinical diagnosis, pathological evaluation, or survival time) is required for model training. As such, it is more challenging to develop deep learning models for tasks in which both input and reference data are difficult to collect, such as the diagnosis of rare diseases. The Cancer Imaging Archive (https://www.cancerimagingarchive.net) currently provides image datasets with appropriate reference labels for relatively common cancers; a similar public database of rare diseases would be helpful to build deep learning models for classifications of these. However, patients’ privacy becomes a more relevant problem in creating such databases.

Next, deep learning models are not necessarily transportable across different hospitals, as indicated by the results described above from Oermann and colleagues showing that deep learning models for detecting pneumonia in chest radiographs showed strong performance with new data from the original training sites but not with external data [3]. When we use deep learning models in actual clinical practice, we must pay attention to how their performance is affected by differences between hospitals, vendors of imaging modalities, and scan or reconstruction conditions. Model training using image data from various settings or patient populations may have the potential to mitigate this problem. However, further investigations would be required to prove this hypothesis. Finally, although a trained model may exhibit high performance in one task such as diagnosis of pneumonia, deep learning in its current forms cannot replace the radiologist’s role in detecting incidental findings such as asymptomatic tumors. This role for radiologists will continue to be invaluable in the era of worldwide population aging, as large numbers of elderly patients have multimorbidity.

In summary, because of the high performance of deep learning in image recognition tasks, the application of this technology to radiological imaging is increasing. If external performance and interpretability improve, AI can be expected to gradually change clinical practice by helping radiologists practice with better performance, greater interrater reliability, and improved workflow for more timely recommendations. Radiologists will be important for labeling training datasets and developing new knowledge from image data, some of which may be inspired by the models. In the clinic, even if current deep learning approaches broadly excel in image interpretation, radiologists will continue to play central roles in the diagnosis of rare diseases and in the detection of incidental findings.

## Abbreviations

AI: artificial intelligence
AUC: area under the receiver operating characteristic curve
CNN: convolutional neural network
CT: computed tomography
MR: magnetic resonance
PET: positron emission tomography
